# Comparing Explanations of Molecular Machine Learning Models Generated with Different Methods for the Calculation of Shapley Values

**DOI:** 10.1002/minf.202500067

**Published:** 2025-03-20

**Authors:** Alec Lamens, Jürgen Bajorath

**Affiliations:** ^1^ Department of Life Science Informatics and Data Science B-IT, LIMES Program Unit Chemical Biology and Medicinal Chemistry Rheinische Friedrich-Wilhelms-Universität Friedrich-Hirzebruch-Allee 5/6 D-53115 Bonn Germany; ^2^ Lamarr Institute for Machine Learning and Artificial Intelligence Rheinische Friedrich-Wilhelms-Universität Bonn Friedrich-Hirzebruch-Allee 5/6 D-53115 Bonn Germany

**Keywords:** approximation methods, compound activity prediction, feature attribution, machine learning, model explanation, Shapley values

## Abstract

Feature attribution methods from explainable artificial intelligence (XAI) provide explanations of machine learning models by quantifying feature importance for predictions of test instances. While features determining individual predictions have frequently been identified in machine learning applications, the consistency of feature importance‐based explanations of machine learning models using different attribution methods has not been thoroughly investigated. We have systematically compared model explanations in molecular machine learning. Therefore, a test system of highly accurate compound activity predictions for different targets using different machine learning methods was generated. For these predictions, explanations were computed using methodological variants of the Shapley value formalism, a popular feature attribution approach in machine learning adapted from game theory. Predictions of each model were assessed using a model‐agnostic and model‐specific Shapley value‐based method. The resulting feature importance distributions were characterized and compared by a global statistical analysis using diverse measures. Unexpectedly, methodological variants for Shapley value calculations yielded distinct feature importance distributions for highly accurate predictions. There was only little agreement between alternative model explanations. Our findings suggest that feature importance‐based explanations of machine learning predictions should include an assessment of consistency using alternative methods.

## Graphical abstract

Comparison of different methods for the derivation of Shapley values. Shown is the schematic workflow for the systematic comparison of different methods to calculate or approximate Shapley values. Various machine learning (ML) models were derived for compound activity predictions. To explain the predictions, several feature attribution methods based upon the Shapley value formalism were applied. The resulting explanations were compared in detail.

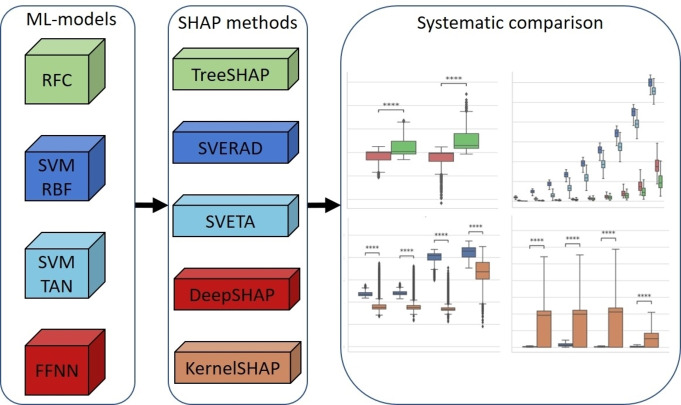

## 1 Introduction

The widespread use of machine learning in science is not only raising hopes for advancing discovery strategies but also awareness of intrinsic limitations of many predictions as a consequence of their black box character [1, 2, 3]. The inability to develop scientific rationales for non‐transparent model decisions causes natural reluctance to depend on them, especially by non‐experts, but also obscures potential model bias or error sources and thus hinders methodological advancements. Moreover, if apparent model accuracy in benchmark settings does not translate into successful prospective machine learning applications, the gap between model promises and acceptance widens. Given that all deep learning models are black boxes, awareness of associated shortcomings in understanding machine learning studies has reached a new level, well beyond similar concerns raised in the past. As a consequence, from several points of view, there is strong interest in methods from explainable artificial intelligence (XAI) to better understand the ways in which machine learning models reach their decisions and to rationalize predictions [4, 5, 6].

XAI covers diverse approaches [4, 5, 6]. For example, various methods have been introduced for determining the importance of features used by machine learning models for predictions. These feature attribution methods often rely on systematic feature masking or perturbation to quantify the influence of individual features on predictions or use local approximation models in given feature spaces. Feature importance analysis can be combined with feature mapping to visualize key features driving predictions of test instances. For deep neural networks, weight gradients can be analyzed to at least partially assess origins of predictions. For transformer models, attention weight matrices or maps might be generated to explore learning processes. Other XAI concepts include counterfactuals that capture minimal feature changes of test instances inverting predictions or contrastive explanations that rely on determining minimally required feature sets for different class label predictions. For graph‐based models, subgraphs determining predictions can be identified or importance weights assigned to nodes or edges. Moreover, methods for quantifying and comparing the probabilities or uncertainties of predictions also aid in model interpretation.

For feature attribution, an approach that has become rather popular in different fields, including chemistry and drug discovery, is the Shapley value formalism that was adapted from cooperative game theory [7]. The Shapley value concept was originally conceived to divide the gain (or payoff) of a game among players forming a team, according to their individual contributions [7]. Shapley values are calculated as follows:

$$\varphi_{f}\left(\nu \right)=\sum_{𝒮\subseteq T\setminus \left\{p\right\}}\frac{\left|𝒮\right|!\left(\left|T\right|-\left|𝒮\right|-1\right)!}{\left|T\right|!}\left(\nu \left(𝒮\cup p\right)-\nu \left(𝒮\right)\right)$$

In this equation, T is the team including all players, 𝒮 is an ordered subset (or coalition) of players, $\nu \left(𝒮\right)$ is the value of the coalition 𝒮 , *p* is a player, and $\varphi_{f}\left(\nu \right)$ the Shapley value of player *p*.

For machine learning, the Shapley value concept is adapted based on the following analogy: Players correspond to features, the game to the prediction of a given test instance, and the gain to the prediction of the test instance after subtracting the mean value of all test set predictions.

In machine learning, Shapley values meet several axioms for consistent feature importance assessment [8]. Moreover, an advantage of the Shapley value approach compared to other feature weighting techniques is that the importance of chemical features that are either present or absent in test instances can be determined.

The Shapley value of an individual player or feature is calculated as the mean marginal contribution to all possible coalitions. For n features or players, coalitions that need to be accounted for correspond to 2^n^ ordered subsets. The adaptation of the Shapley value concept for machine learning faces two major challenges. First, given the requirement to determine the marginal contribution for all coalitions, the calculation of exact Shapley becomes computationally infeasible for large feature sets that are typically used. Second, machine learning models are unable to make predictions using subsets of features they were trained on. Thus, to determine the contribution of a given coalition, all features not contained in the coalition must be randomized or sampled from a marginal distribution to quantify the deviation from the original prediction. To address both of these challenges, approximation methods have been introduced to facilitate Shapley value calculations for machine learning models, many of which rely on feature perturbation and local approximations [8], which might give rise to inconsistencies in explanations. Therefore, we compare different approaches for the approximate or exact calculation of Shapley values for compound activity predictions using different machine learning models to evaluate feature attribution characteristics and assess the consistency of explanations in a quantitative manner.

## 2 Methods

### 2.1 Compounds and Activity Data

Compound activity classes were extracted from CHEMBL (version 33) [9], a manually curated database containing ~2.4 million bioactive molecules and drugs collected from the medicinal chemistry literature. To omit compounds that were much larger than typical small molecular drugs or had questionable activity annotations, any compounds with a molecular mass >1000 Da or with labels “inactive”, “inconclusive”, “not active”, “potential author error” or “potential transcription error” were removed. In addition, only compounds with a numerically specified standard activity measurement were considered including inhibition (K_i_) and dissociation (K_d_) constants, which quantify the binding affinity of a compound to a target, and the compound concentration at halfmaximal (50%) inhibition 50% of the activity (IC_50_). Furthermore, the measurements were required to be exactly quantified using the standard relationship (“=”), ranging from 10 μM (lowest potency) to 10 pM (highest), in order to avoid consideration of imprecise measurements. In addition, activity had to be measured in a direct interaction assay with a single target protein (referred to in ChEMBL as target relationship type “D”), that is, a biochemical target‐specific assay, rather than any cell‐based assays, in which multiple (unknown) targets might contribute. Moreover, activity measurements were required to be reported at the highest ChEMBL assay confidence score of 9. Finally, compounds with potential activity against undesired targets such as serum‐albumin or drug‐metabolizing cytochrome P450 isoforms and potential assay interference compounds were also removed using publicly accessible tools and filters [10, 11]. ChEMBL compounds are organized as target‐based compound activity classes (i. e., sets of compounds with activity against a particular biological target). To obtain activity classes with large numbers of active compounds for machine learning, a final count of more than 1000 qualifying active compounds was required following data curation, as described above, leading to the selection of the 10 activity classes for different proteins, reported in Table 1. With the exception of two carbonic anhydrase isoforms, these activity classes covered functionally distinct targets. Each of the 10 activity classes represented a unique data set for binary compound classification (that is, active versus inactive). Therefore, compounds active against an individual target were supplemented with an equal number of compounds randomly selected from ChEMBL after exclusion of these activity classes and used as presumed inactive compounds for this class. Thus, for binary classification of each of the 10 unique data sets using machine learning, as detailed below, two classes of compounds of identical cardinality were used with class labels “active” or “inactive” against the target protein of this data set.

**Table 1:** Activity classes.

Protein target	# Active compounds
Carbonic anhydrase 1	3858
Carbonic anhydrase 9	3779
Vascular endothelial growth factor receptor 2	2523
Acetylcholinesterase	2068
Histone deacetylase 1	2028
Dipeptidyl peptidase 4	1740
Bifunctional epoxide hydrolase 2	1442
Hepatocyte growth factor receptor	1414
Beta‐secretase 1	1380
Cholinesterase	1305

### 2.2 Molecular Representation

Each selected compound was represented using the folded 2048‐bit version of the extended connectivity fingerprint with a bond diameter of 4 (ECFP4) calculated with RDKit [12, 13]. ECFP fingerprints capture layered atom environments, as detailed in the original reference [12]. Accordingly, the environment of an atom is defined by sets of neighboring atoms falling into individual layers within a specified bond diameter. For instance, a bond diameter of 4 (corresponding to a bond radius of 2) means that layers capturing all atoms that are one and two bonds away from the root atom are recorded in the fingerpint. Among possible ECFP variants, ECFP4 is most widely used for machine learning because of its stable performance, while avoiding the inclusion of increasingly redundant atom environments for large diameter settings [14, 15].

### 2.3 Machine Learning Models

For each of the 10 activity class data sets, binary classification models were generated using different machine learning methods to systematically distinguish between active (positive) and inactive/random (negative) compounds. Machine learning methods of different computational complexity included random forest classifiers (RFC) [16, 17], feed‐forward neural networks (FFNN) [18], and support vector machines (SVM) [19] with a radial basis function (RBF) kernel (SVM_RBF) or Tanimoto kernel [20] (SVM_TAN). For each data set and model, 10‐fold cross‐validation was carried out to obtain 10 independent classification trials by partitioning each activity class into 90% training and 10% test compounds using the ‘stratisfied shufflesplit’ function of scikit‐learn [21]. Additionally, the cross‐validation scheme was used to optimize model hyperparameters for each independent trial based on the training data following 10‐fold (90% vs. 10%) data partition to generate training and validation sets (exclusively for hyperparamter optimization).

RFC and SVM models were implemented using scikit‐learn.^[21]^ For hyperparameter optimization with the F1‐score [22] as a metric, the grid search function of scikit‐learn was used. For RFC models, the minimal number of samples for a leaf node (1, 2, 5, 10), minimal number of leaves required for a split (2, 3, 5, 10), and the number of decision trees (25, 50, 100, 200, 400) were optimized. For SVM_RBF models, parameters C (0.001, 0.005, 0.01, 0.05, 0.1, 0.5, 1, 10, 100, 10000) and gamma (0.0001, 0.001, 0.01, 0.1, 1, 10, 100) were optimized. For SVM_TAN models, the C parameter was optimized accordingly.

A simple FFNN was implemented using Tensorflow [23] comprising a single hidden layer of size 256 and the ReLU activation function, Adam optimizer [24], and a batch size of 16. As a loss function, binary cross entropy was used. The model was trained for a maximum of 400 epochs, with an early stop criterion if validation loss did not change for 20 epochs. As a hyperparameter, the initial learning rate was optimized (0.01, 0.001, or 0.0001).

For the evaluation of model performance using different measures, active compounds were labeled “positive” and inactive compounds “negative”. Accordingly, possible prediction outcomes are categorized as follows:

1. Positives that are correctly predicted: true positives (TP),
2. Positives that are incorrectly predicted: false negatives (FN),
3. Negatives that are correctly predicted: true negatives (TN),
4. Negatives that are incorrectly predicted: false positives (FP).

Using these categories, the classification accuracy of all models was quantified using five performance measures:

1) Precision is interpreted as the ability of a model to correctly classifiy positive instances and defined as:

$$Precision=\;\frac{\mathrm{TP}}{TP+FP}$$

Here, TP and FP are considered. This measure only considers positives instances and ranges from 0 to 1, where a value of 1 reflects perfect predictions of positive test instances.

2) Recall, also known as the true positive rate (TPR), quantifies the ability of a model to identify all positive instances and is calculated as:

$$\;Recall=\;\frac{\mathrm{TP}}{TP+FN}$$

TP and FN are considered. Recall is important for assessing whether all true positive instances are identified, without considering false positives. It ranges from 0 to 1, with a value of 1 indicating that each positive instance was classified correctly.

3) Balanced accuracy (BA) [25] defines the arithmetic mean of the sensitivity (recall) and specificity. It is applied to determine the rate of correct TP and TN predictions, referred to as TPR and TNR, respectively, and is defined as:

$$BA=\;\frac{1}{2}\left(TPR+TNR\right)$$

Here specificity (TNR) measures the fraction of correctly identified TN instances over all predictions of negative instances. TPR is identical to the recall metric, as defined above. BA ranges from 0 to 1, with a value of 1 indicating perfect predictions.

4) The F1‐score (F1) [22] is defined as the harmonic mean of precision and recall. Thus, it consititutes a balanced measure of the trade‐off between precision and recall, defined as:

$$F1=2\;\times \;\frac{\mathrm{TP}}{2TP+FP+FN}$$

TP, FP, and FN are considered. F1 has a value range from 0 to 1, with a value of 1 indicating perfec predictions.

5) Matthew′s Correlation Coefficient (MCC) [26], a balanced measure accounting for the confusion matrix, is unaffected by data imbalance and defined as:

$$MCC=\;\frac{TP\times TN-FP\times FN}{\sqrt{\left(TP+FP\right)\left(TP+FN\right)\left(TN+FP\right)\left(TN+FN\right)}}$$

TP, FP, TN and FN are considered. MCC ranges from −1 to 1, with 1 indicating fully accurate predictions, 0 random predictions, and −1 inverted (completely inaccurate) predictions.

### 2.4 Model Explanation

#### 2.4.1 Shapley Value‐Based Methods

The Shapley additive explanations (SHAP) approach [27] represents the most popular algorithmic approximation of Shapley value calculations in cheminformatics and medicinal chemistry [8, 28, 29]. SHAP expresses the Shapley value formalism as an additive feature attribution approach using a linear local approximation model with a specifically designed kernel function to estimate Shapley values via linear regression [27]. Accordingly, the original SHAP methodology is also referred to as KernelSHAP, as used in the following. While KernelSHAP is model‐agnostic, algorithm‐specific SHAP variants have been introduced including TreeSHAP [30] for decision tree methods and DeepSHAP [31] for deep neural networks that build upon the underlying structure of the algorithms to further increase the efficiency and accuracy of the approximations. All SHAP variants have in common that features missing in coalitions are sampled from a training data background distribution. KernelSHAP and DeepSHAP are not applicable to sample coalitions exhaustively, given their local approximations of Shapley values. For standard machine learning methods, non‐approximated direct calculation of exact Shapley values is currently possible for SVM models using binary features and either the RBF or Tanimoto kernel with the Shapley value‐expressed Tanimoto similarity (SVETA) [32] and Shapley value‐expressed radial basis function (SVERAD) [33] methods, respectively. Additionally, TreeSHAP with interventional feature perturbation calculates exact Shapley values under the assumption of feature independence, making use of the specific architectural features of decision trees [34, 35]. Moreover, most approximation methods for Shapley values are based on the marginal expectation where the average model output is considered when a subset of features in a given coalition is constant and remaining features are varied based on their marginal distributions [36]. Assuming feature independence, this yields an approximation of the contribution of the feature in isolation. This assumption might lead to non‐intuitive explanations when strong correlations in the data are observed. Alternatively, the conditional expectation can be used. In this case, the average output of the model is calculated for coalitions when a subset of features is held constant and the remaining values are drawn from the conditional (observational) distribution [36]. Thus, the contribution of a feature is considered in the context of observed feature combinations, accounting for feature dependence. This often requires further approximations to reduce computational complexity. Additionally, the conditional approach enables attribution of importance to irrelevant features. The choice of the marginal or conditional expectation is considered to depend on the preference to be more dedicated to the model or the data, respectively [37].

Irrespective of the method of handling missing values in coalitions, the calculation of exact Shapley requires exhaustively accounting for contributions across all coalitions. Synthetic (hypothetical) data examples for the exact calculation of Shapley values using limited feature numbers and complete coalitions have been reported previously [32, 33], highlighting the varying degree to which SHAP variants approximate exact Shapley values, while SVERAD and SVETA produced identical feature attributions to exact calculations. These observations can also be taken into consideration when comparing different calculation and approximation methods.

**Figure 1:**
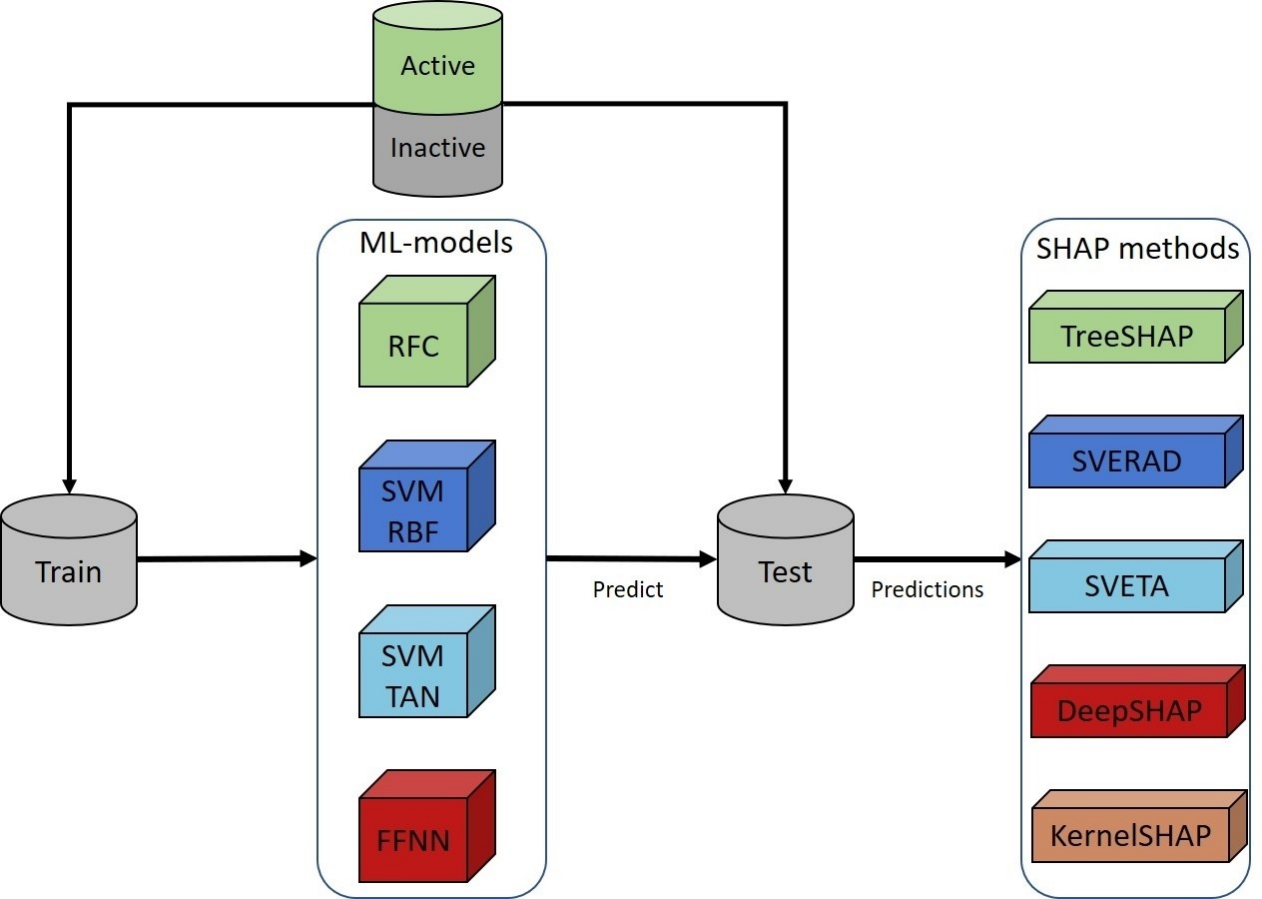
Analysis workflow. Shown is a summary of the analysis protocol.

Figure 1 summarizes the analysis protocol. Following compound data curation, different ML models were derived to distinguish active from inactive compounds, followed by comparative analysis of exact Shapley and corresponding SHAP values for feature attribution.

Given the availability of the three SHAP variants, SVETA, and SVERAD, our comparative analysis of Shapley value‐based model explanations was carried out using the model combinations reported in Table 2.

**Table 2:** Shapley value‐based explanation methods.

Machine learning method	Explanation methods
RFC	TreeSHAP (interventional) KernelSHAP
FFNN	DeepSHAP KernelSHAP
SVM_RBF	SVERAD KernelSHAP
SVM_TAN	SVETA KernelSHAP

#### 2.4.2 Cumulative SHAP/Shapley Value Analysis

Given that explanations are computed for individual test instances, cumulative SHAP/Shapley value analysis for all features present and absent in test instances was carried out by summing importance values of individual features over all correctly predicted active or inactive test compounds. As stated above, a key aspect of the SHAP/Shapley value formalism is that the contributions of representation features to a prediction can be quantified that are either present or absent in a test compound [7, 8, 27]. In the fingerprint of a given test compound, the bit corresponding to a particular feature that is absent in this compound is set to 0. Cumulative SHAP/Shapley value analysis made it possible to globally assess feature value distributions and their differences.

#### 2.4.3 Statistical Measures

Contributions of feature importance values to predictions were statistically assessed in different ways.

The Gini coefficient [38] was applied as a measure of the dispersion of feature importance values for individual compounds. Accordingly, a large Gini coefficient indicates that a limited number of features largely determines a model decision. Conversely, a small Gini value indicates that a large number of features contribute in a comparable manner to the model decision. For values ordered by magnitude, the Gini coefficient is calculated as follows:

$$G=\;\frac{\sum_{i=1}^{n}\left(2i-n-1\right)X_{i}}{n\sum_{i=1}^{n}X_{i}}$$

Here, n represents the total number of values, X the observed value, and i the rank of the value in ascending order. The formula only applies to non‐zero values.

*Compacity* [39] was calculated to quantify feature numbers required for individual predictions. When feature importance values are arranged in descending order, compacity is defined as follows:

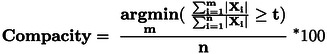




Compacity refers to the number of feature attribution values m that contribute to the cumulative sum ($\sum_{i=1}^{m}\left|X_{i}\right|$ ), reaching a predefined threshold t of the absolute sum for all feature attributions for an instance ($\sum_{i=1}^{n}\left|X_{i}\right|$ ) expressed as a percentage of the total number of features n . Accordingly, compacity determines how many features were required for a given model decision.

As a measure of *consistency* of feature contributions produced by all methods, the standard deviation of importance values of all features was calculated. First, the SHAP/Shapley values for each test instance were normalized by dividing each value by the absolute sum of all values. Then, the standard deviation was calculated for each feature across all test instances with a non‐zero value.

As a measure of *correlation* between the feature importance values produced by different methods, Pearson′s correlation coefficient was calculated, with values ranging from −1 to 1, indicating a perfect inverse linear correlation or perfect linear correlation between two value populations, respectively. Correlation was determined for test instances from all activity classes across all trials and considering either all, present, or absent features.

*Faithfulness* [40, 41] was calculated to determine if feature importance values reflected the marginal contribution of the feature to a prediction:

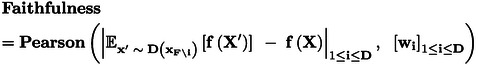




In other words, faithfulness measures whether or not given feature importance values (regardless of how they were obtained) accurately reflect the relative contributions of features to a prediction. It is obtained as the Pearson correlation coefficient between the feature importance vector w and the marginal contribution for each feature i to the model prediction f for instance X . As an adaptation to the binary feature fingerprints used to represent chemical structure, the marginal contribution of each feature was determined by inverting the bit setting from 1 to 0 or vice versa.

*Remove‐and‐retrain* (ROAR) [42] analysis was carried out to determine if the different methods were able to globally identify features important for the correct prediction of test instances. Initially, the SHAP/Shapley value feature importance matrices obtained from each method variant were used to identify the most important features. SHAP/Shapley values for each test instance were normalized by dividing each value by the absolute sum of all SHAP/Shapley values for a test instance. Then, the absolute sums of the values of each feature across all test instances were ranked in ascending order to determine most important features. After identifying the top‐ranked n features for each method, the least important features were iteratively removed (n=100, 1000, 1500, 1700, 1800, 1900, 1950, 2000, 2023, 2038, 2043) and the models were retrained using the reduced feature sets. The resulting models were then used to predict the test set with the identical reduced feature set. As a control, features were randomly removed from the feature set. To measure model performance, MCC was calculated.

As a measure of statistical significance, the Wilcoxon signed‐rank test [43] with Holm‐Bonferroni correction [44] was carried out.

### 2.5 Data and Code Availability

All data and code used for our analysis are freely available via the following link: https://uni‐bonn.sciebo.de/s/XBiuWn0NKkE5qhE.

## 3 Results

### 3.1 Machine Learning Predictions

**Figure 2:**
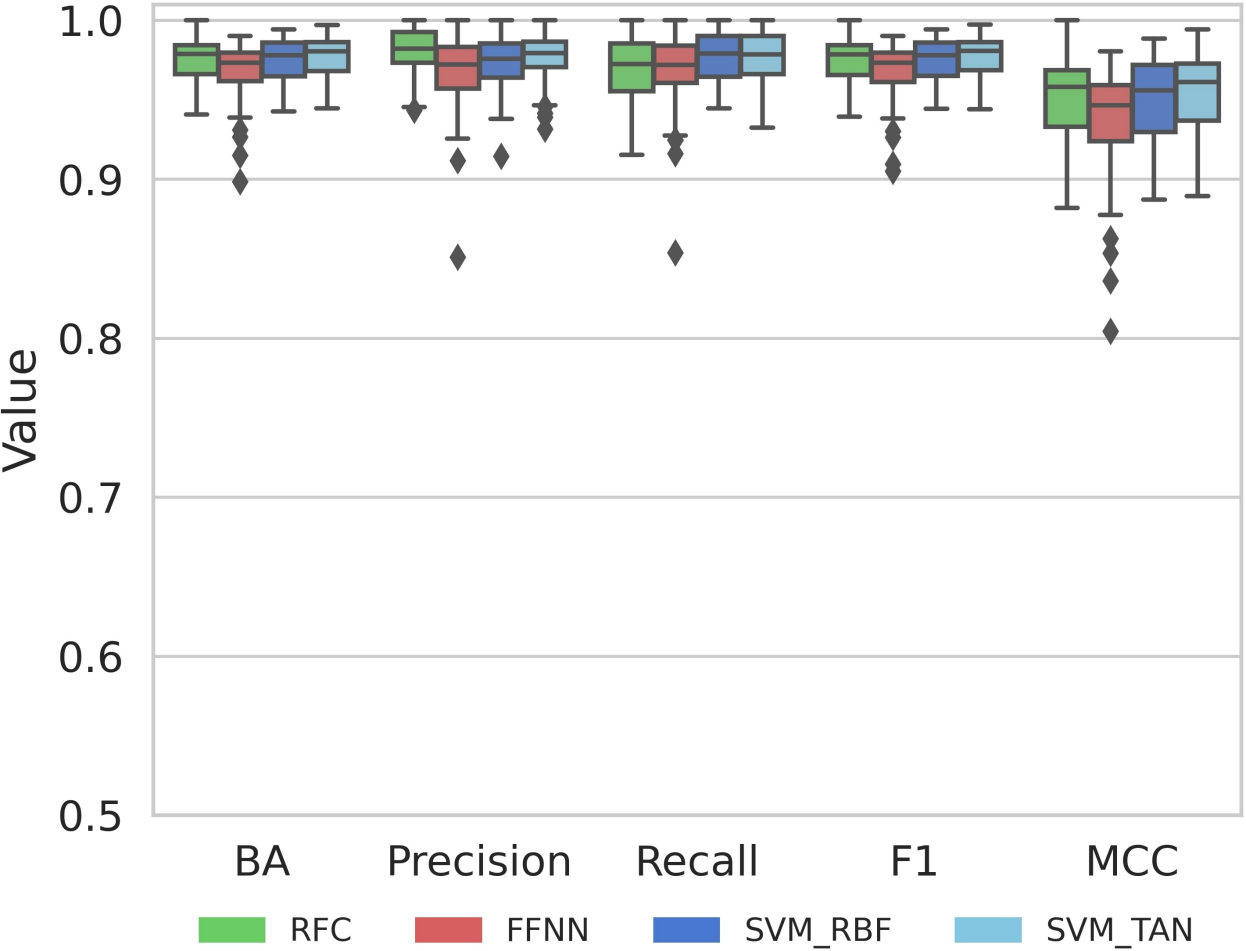
Prediction accuracy. Boxplots report the performance of different compound classification models in test set predictions for all 10 activity classes over 10 independent trials.

As a basis for comparative model explanation analysis, standard compound activity predictions using different classification models were carried out. Figure 2 summarizes the results over 10 activity classes using different performance measures.

On the basis of all performance measures, consistently highly accurate predictions were obtained, with median accuracy approaching 100%, narrow value distributions (reflecting the stability of independent predictions), and only limited numbers of statistical outliers. In addition, high median MCC values of ~0.95 were obtained. Accordingly, these nearly ideal activity predictions over different classes provided a solid basis for a comparative global analysis of model explanations. Therefore, the results for all activity classes were combined.

### 3.2 Cumulative Feature Importance Analysis

**Figure 3:**
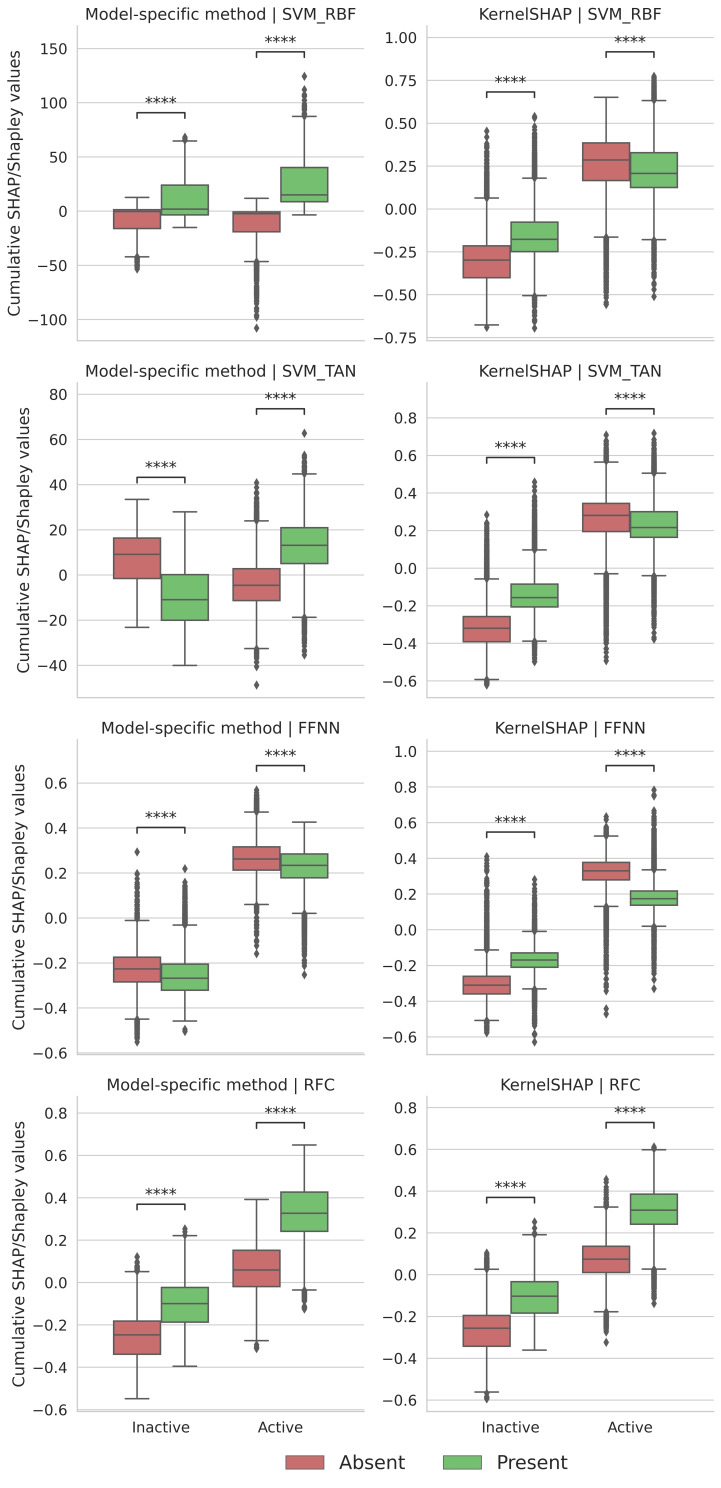
Cumulative SHAP/Shapley value analysis. Boxplots report the relative contribution of features present or absent in test compounds from all activity classes to correct predictions using different explanation methods. Each data point represents the sum of SHAP/Shapley values of all features present or absent in a single test compound. “Model‐specific method” refers to SVERAD (for SVM_RBF), SVETA (SVM_TAN), DeepSHAP (FFNN), and TreeSHAP (RFC). For SVERAD and SVETA, Shapley values are calculated and reported on a logit (natural logarithmic) scale. Statistical significance of differences between corresponding value distributions for present and absent features was assessed using a Wilcoxon test with a Holm‐Bonferroni correction. Increasing statistical significance is indicated as follows: Not significant (ns): 5.00e–02<p<=1.00e+00, * 1.00e–02<p<=5.00e–02, ** 1.00e–03<p<=1.00e–02, *** 1.00e–04<p<=1.00e–03, **** p<=1.00e–04.

For each machine learning method, model‐agnostic KernelSHAP calculations were carried out in comparison to a model‐specific SHAP/Shapley value method. For each correctly predicted active or inactive test compound, importance values for all present and absent features were determined. Then, feature importance values were summed over all test compounds in which they occurred over the activity classes. The results of cumulative SHAP/Shapley value analysis are reported in Figure 3. Positive and negative values indicate contributions to the prediction of activity and inactivity, respectively. For RFC, KernelSHAP and TreeSHAP produced corresponding results. Features present in active test compounds contributed to correct predictions while absent features made, for the most part, only marginal contributions. In addition, features present and absent in inactive compounds contributed to their correct prediction, with absent features making larger contributions than observed for active compounds. For FFNN, KernelSHAP and DeepSHAP produced similar value distributions for active compounds, with absent features making larger contributions to correct predictions than present features, whereas relative contributions of features present or absent in active compounds to their predictions were reversed. For SVM_RBF, Shapley values calculated with SVERAD for features present or absent in inactive compounds made only marginal contributions to incorrect and correct predictions, respectively. Features present in active compounds contributed to correct predictions while absent features made marginal negative contributions. In this case, KernelSHAP produced different results. Here, features present or absent in active and inactive compounds contributed to their correct predictions. KernelSHAP also yielded comparable value distributions for SVM_TAN. However, relative feature contributions based on Shapley values calculated with SVETA differed. In this case, features absent in inactive compounds opposed their correct prediction whereas present features supported it. Corresponding contributions were obtained for features absent or present in active compounds. Hence, SVERAD and SVETA calculations indicated a notable influence of the kernel function on SVM classification, different from KernelSHAP.

The comparison in Figure 3 shows that relative feature contributions varied for the different machine learning methods, as might be expected. Moreover, relative feature contributions assessed using KernelSHAP and corresponding model‐specific approaches were only closely corresponding for RFC, but not FFNN, SVM_RBF, and SVM_TAN. On the basis of these observations, differences between importance value distributions and contributions to correct predictions were further analyzed.

### 3.3 Comparison of Explanation Methods

**Figure 4:**
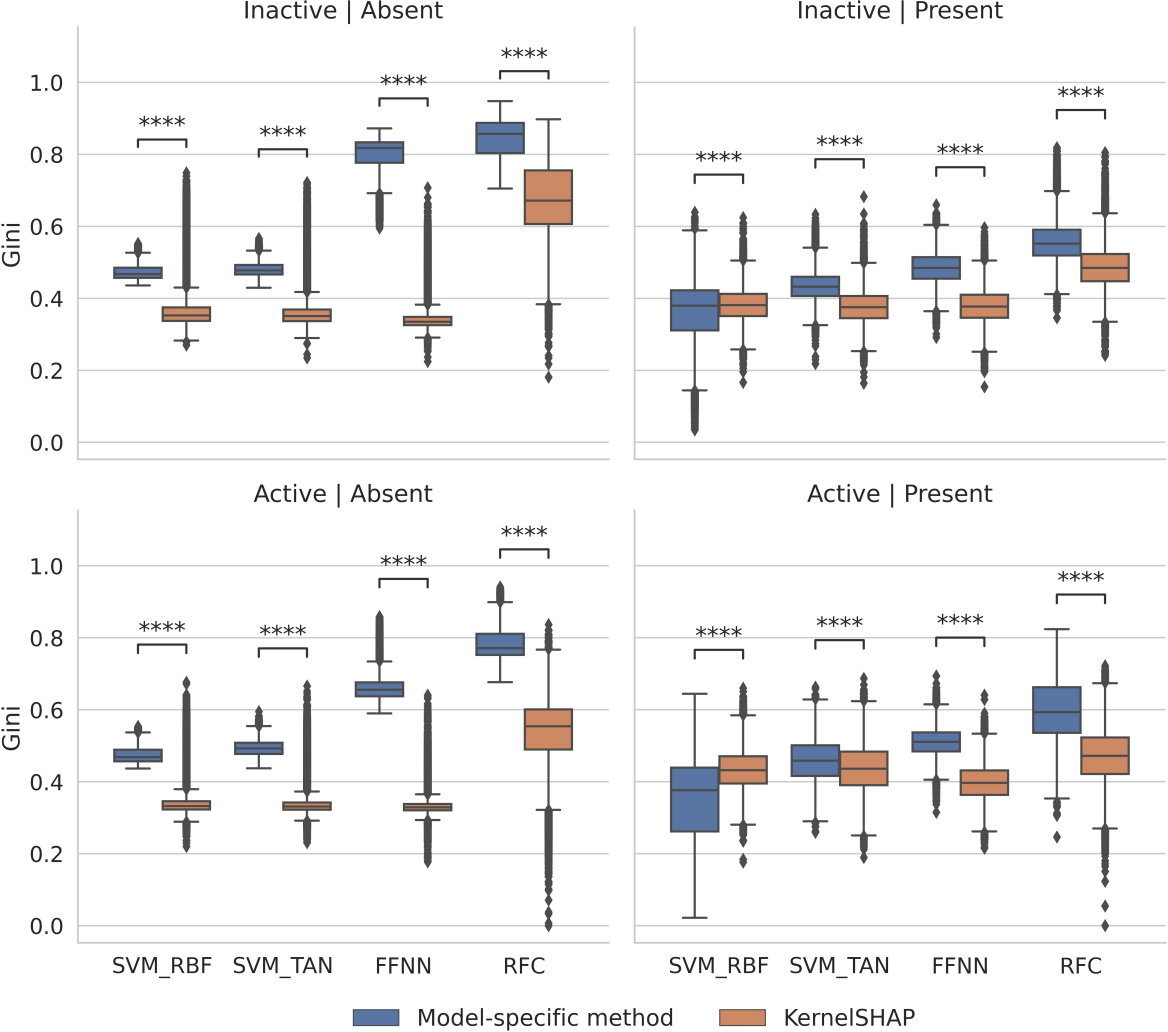
Gini coefficients. Boxplots report the distributions of Gini coefficients of SHAP/Shapley values for individual test compounds (each data point represents a single compound). The Gini coefficient was calculated for test instances across all activity classes and trials. Results are shown for different feature categories. For example, “Active I Present” (lower right) designates features present in correctly predicted active test compounds. Model‐specific methods are designated according to Figure 3. Statistical significance of differences between corresponding value distributions is assessed and indicated according to Figure 3.

We first calculated Gini coefficients to identify features determining the highly accurate predictions with different classification methods. The results in Figure 4 show that Gini coefficient distributions for KernelSHAP compared to model‐specific methods consistently displayed statistically significant differences. For features absent in active and inactive compounds, coefficient values for KernelSHAP differed greatly, leading to value distributions that were largely determined by statistical outliers. However, model‐specific methods mostly produced larger values and better defined distributions, consistently resulting in much larger median values than observed for KernelSHAP. For features present in active and inactive compounds, similar trends were observed, but differences between Gini coefficient medians were smaller. Here, an exception was detected for for SVM_RBF where KernelSHAP produced slightly larger median values than SVERAD. Overall, a consistent trend was observed that model‐specific methods prioritized fewer features making largest contributions to predictions than KernelSHAP. These features can be considered as “key features” for predictions.

**Figure 5:**
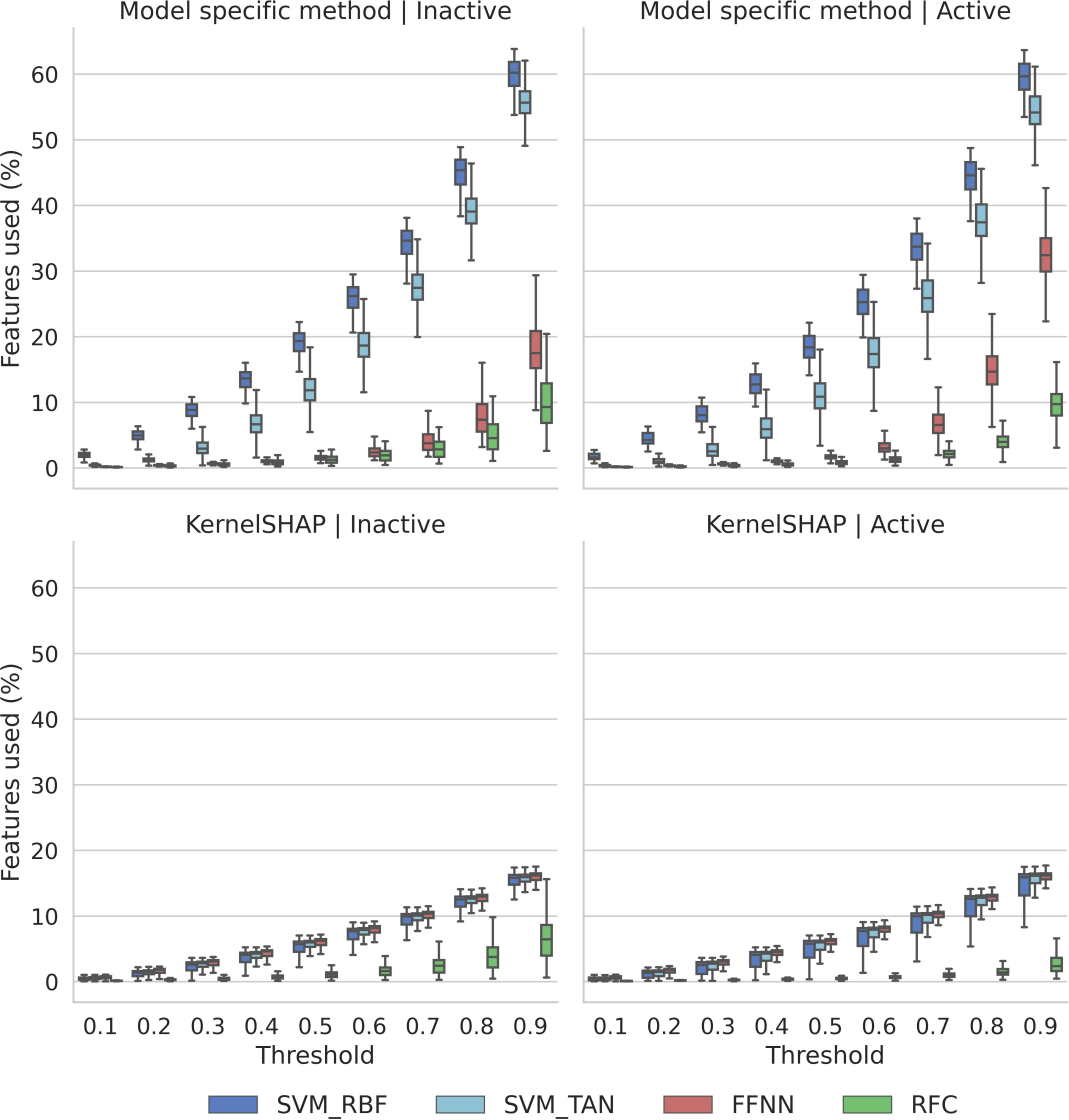
Compacity. Boxplots report the distributions of SHAP/Shapley values of the percentages of all present and absent features of individual test compounds required to reach increasing compacity thresholds. Results are shown for test instances from all activity classes and trials.

These observations were further investigated by compacity analysis, quantifying feature contributions to predictions in a different way. While large Gini coefficients indicated the presence of small numbers of key features for predictions, in this case, the fraction of all features was determined that contributed to increasing proportions (compacity thresholds) of the absolute sum of all (positive and negative) feature contributions to correct predictions. Thus, compacity more broadly accounted for contributions of feature subsets, rather than emphasizing the presence of key features. The results are reported in Figure 5.

For both inactive and active compounds and all classifiers except RFC, KernelSHAP produced very similar results. Small feature subsets were sufficient to meet increasing compacity thresholds. Ultimately, less than 20% of all features were required to account for 90% of the total feature contributions, with very narrow value distributions across all test compounds. For RFC, both KernelSHAP and TreeSHAP produced even smaller feature subsets, with at most 10% of all features required to meet the 90% compacity threshold. For the other three model‐specific methods, a different picture emerged. For inactive compounds, feature subsets produced with DeepSHAP were similar in size to TreeSHAP, except for the 0.9 threshold when a notable increase was observed. However, for active compounds, the size of DeepSHAP feature subsets further increased beginning at the 0.7 threshold and more than 30% of all features were required to account for 90% of the total feature sum. Strikingly, for SVERAD and SVETA, there was a nearly exponential increase in the size of feature subsets across the entire threshold range for both inactive and active compounds, in contrast to the other explanation methods, with a final median size of 60% (SVERAD) or close to 60% (SVETA) of all features.

Taken together, the results of Gini coefficient and compacity analysis revealed very different relative feature contributions to accurate predictions by KernelSHAP and model‐specifc explanation methods. For example, for KernelSHAP, consistently fewer than 20% of all features were sufficient to account for most feature contributions, however, in the absence of prominent key features. By contrast, TreeSHAP strongly prioritized key features making largest contributions to predictions, but only very few other features made notable contributions. On the other hand, SVERAD/SVETA also produced more key features than KernelSHAP, but many other features made small contributions to the predictions, as revealed in Figure 5.

### 3.4 Consistency and Correlation of Feature Importance Values

**Figure 6:**
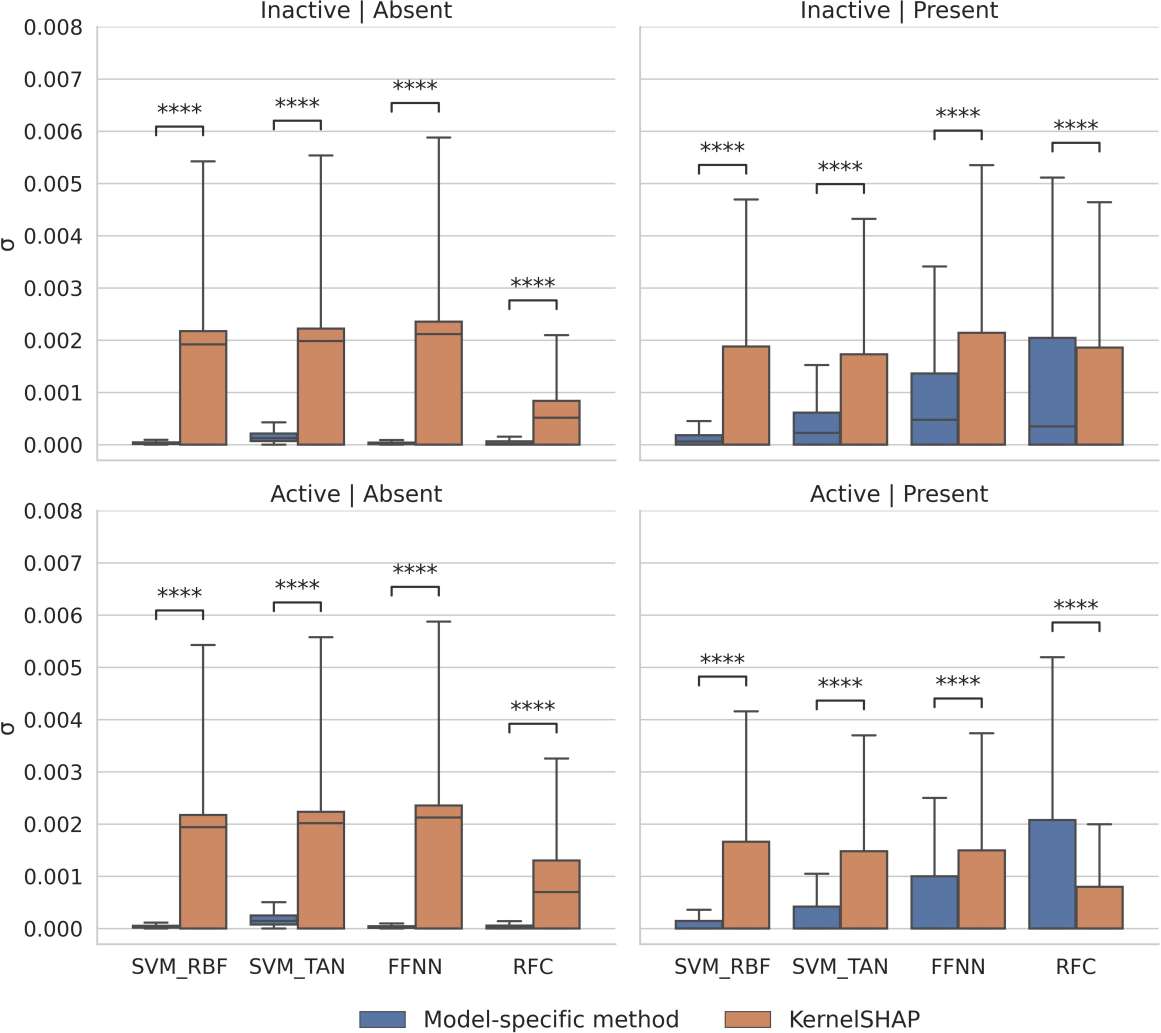
Consistency of feature importance values. Boxplots report the standard deviations of normalized SHAP/Shapley values for all features across test compounds. The presentation is according to Figure 3.

Next we analyzed the consistency of feature contributions by determining the standard deviations of values of each contributing feature across all test compounds, as shown in Figure 6.

For features absent in inactive or active compounds, standard deviations for model‐specific methods were consistently close to zero, but larger for KernelSHAP. For features present in inactive or active compounds, standard deviations slightly increased for SVERAD/SVETA and more strongly for DeepSHAP and TreeSHAP, while the deviations consistently decreased for KernelSHAP. The strongest relative increase was detected for TreeSHAP where standard deviations exceeded KernelSHAP for both inactive and active compounds. Differences between all corresponding value distributions were statistically significant.

**Figure 7:**
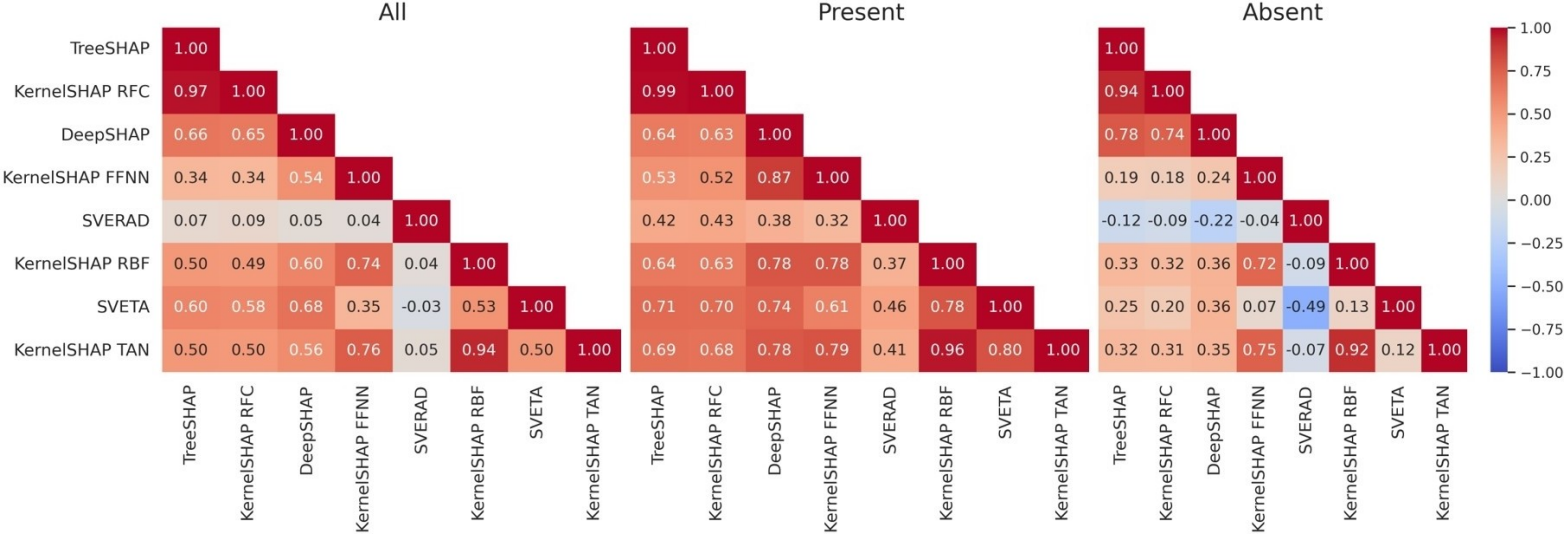
Feature importance value correlation. Heatmaps report Pearson′s correlation coefficients for SHAP/Shapley values of all, present, or absent features calculated with different methods. Feature importance values were compared for test instances from all activity classes and independent trials.

Furthermore, very different degrees of feature correlation were observed for different combinations of methods, as shown in Figure 7. For most combinations, correlation was low for present or absent features, with only few exceptions for all features including KernelSHAP/TreeSHAP (0.97), KernelSHAP for SVM_TAN/SVM_RBF (0.94), and KernelSHAP for SVM_TAN/FFNN (0.76) and SVM_RBF/FFNN (0.74). By contrast, feature correlation between model‐specific methods was generally low or absent.

**Figure 8:**
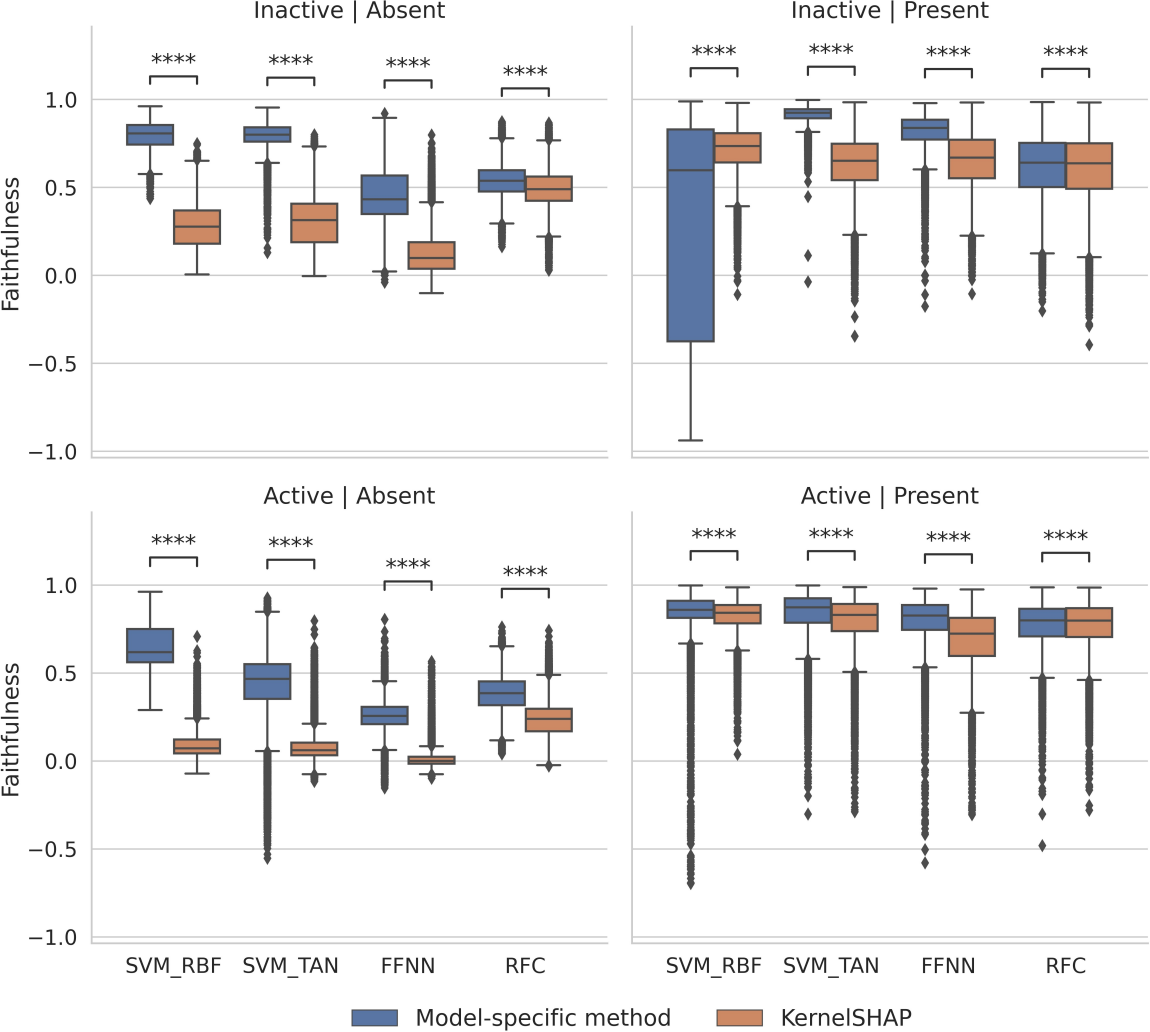
Faithfulness. Boxplots report the distributions of Pearson′s correlation coefficients between the feature importance values and marginal contributions of each feature to predictions. Comparisons were carried out for features of test compounds from all activity classes and trials. The presentation is according to Figure 3.

In addition, the correlation between feature importance values and marginal contributions to predictions was also determined. Figure 8 shows the results of faithfulness analysis.

Boxplots report the distributions of Pearson′s correlation coefficients between the feature importance values and marginal contributions of each feature to predictions. Comparisons were carried out for features of test compounds from all activity classes and trials. The presentation is according to Figure 3.

For features present in active compounds, comparably strong correlation was observed for all approaches (although the value distributions contained numerous statistical outliers with low or no correlation). For features present in inactive compounds, strong correlation was only detected for SVETA while all other median correlation values were lower than for features present in active compounds. Here, an exceptionally broad value distribution was observed for SVERAD, ranging from detectable positive to negative correlation for test instances. For features absent in inactive compounds, correlation was generally low for model‐specific methods and absent for KernelSHAP. For features absent in active compounds, strong correlation was detected for SVERAD and SVETA and correlation values were lower for KernelSHAP compared to corresponding model‐specific methods. Thus, faithfulness analysis also revealed the presence of very different correlation patterns.

### 3.5 Feature Removal

**Figure 9:**
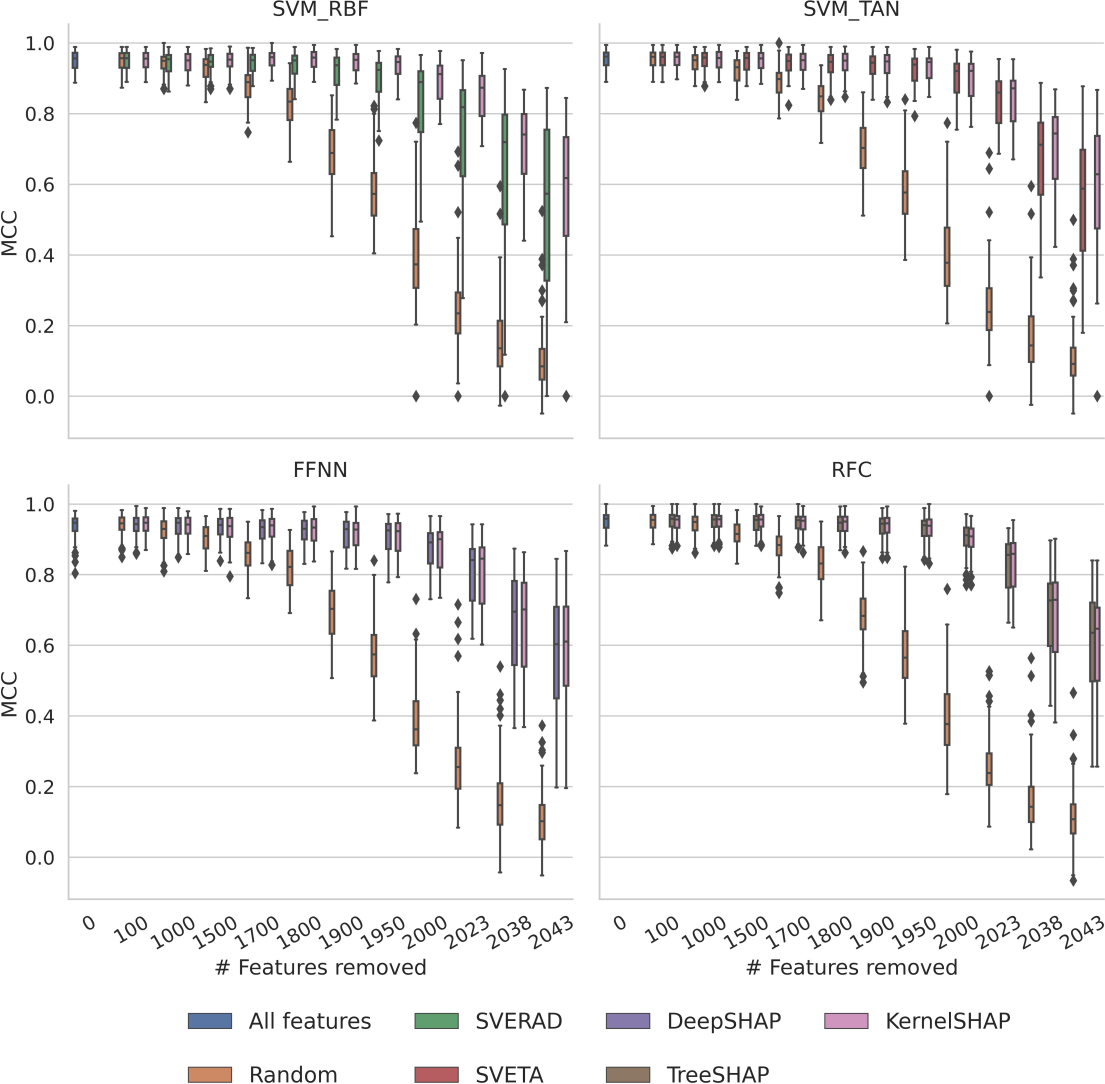
Remove‐and‐retrain analysis. Boxplots report the performance of models trained and tested on iteratively reduced feature sets based on MCC values. In each step, the n least important features determined using alternative explanation methods were removed. As a control, equally sized randomly selected feature subsets were removed and corresponding models retrained.

Finally, we also investigated the influence of iterative removal of features ranked by increasing importance on the predictions. Figure 9 summarizes the results of ROAR analysis. For all machine learning and explanation methods, computed feature importance ranking was indicated to be meaningful because prediction accuracy rapidly and consistently decreased by random feature removal when feature importance increased. By contrast, predictions were much less affected by removal of features in the order of increasing importance and remained stable for smaller feature subsets until 2000 or more features were removed. Prediction accuracy of retrained models was overall similar when feature importance was assessed with KernelSHAP and corresponding model‐specific methods. These findings also indicated that the different methods identified and highly ranked features determining the predictions, although relative feature importance assessed using different methods significantly varied, as shown above. Notably, only ~50 and five to 10 of the most important features were ultimately required to produce moderately or weakly predictive machine learning models, respectively, consistent with the findings reported in Figure 5 and earlier observations [45].

## 4 Conclusions

In this work, we have set out to assess the consistency of feature importance‐based model explanations. Therefore, we have systematically determined and analyzed feature importance distributions based on SHAP/Shapley values for compound activity predictions across different targets. While feature importance is quantified for individual predictions following the Shapley value concept, feature importance was assessed at a global level over a large number of predictions to gain some general insights into feature value distributions. Therefore, cumulative SHAP/Shapley value analysis was initially carried out. Then, the resulting distributions were analyzed and compared in detail. For SVM, RFC, and FFNN classifiers, the model‐agnostic KernelSHAP approach was compared to a model‐specific methodological variant including two approaches for the exact calculation of Shapley values for SVMs using different kernel functions and one approach for the exact calculation of Shapley values for decision tree methods. For different machine learning methods, relative feature contributions varied, as one might expect. However, model‐agnostic and ‐specific explanation methods also produced varying feature importance distributions for predictions using the same models in three of four cases. Significant agreement was only observed for KernelSHAP and TreeSHAP in RFC (which are closely related), but no other combination. The non‐deterministic sampling procedure underlying the SHAP approximation and absence of feature dependence assessment might be expected to lead to inconsistencies of explanations based on SHAP values [46], although the potential magnitude of such inconsistencies would be difficult to forecast.The observed instability for KernelSHAP likely results from the way in which coalitions are sampled where multiple executions of the sampling procedure for the same instance can produce different outcomes [46]. In our analysis, there was agreement between KernelSHAP and TreeSHAP, but not between KernelSHAP and DeepSHAP or KernelSHAP and SVETA/SVERAD, uncovering deviations of a large magnitude. The application of different statistical measures consistently yielded significant differences between corresponding feature value distributions, revealing distinct feature contributions to the predictions. Notably, all SHAP/Shapley value calculation variants identified features determining predictions, as confirmed by retraining and testing classification models after iterative removal of ranked features. However, the differences in relative contributions of features present or absent in active and inactive test compounds resulted in distinct feature importance‐based explanations, although all method variants were based upon the Shapley value concept. Several factors might cause the observed differences such as the limited convergence of approximation methods [33] and the stochastic nature of the approximation procedures, suggesting the need for further algorithmic comparisons of SHAP variants and their sampling characteristics [46] and convergence behavior. However, given the apparent differences, it currently is essentially impossible to prioritize an individual approach such as model‐agnostic KernelSHAP. For XAI, an immediate implication of our analysis is that model explanation should not rely on singular approaches. Instead, different methods should be applied to evaluate consistency of or differences between alternative explanations. While our current analysis is confined to compound activity predictions, for feature attribution analysis using Shapley values, the parallel use of a model‐agnostic and ‐specific adaptation is also suggested for other applications. This will make it possible to evaluate alternative model explanations, assess their (in)consistency, and judge how to proceed with these explanations.

## Conflict of Interests

The authors declare no conflicts of interest.

5

## Data Availability

The data that support the findings of this study are available in ChEMBL at https://www.ebi.ac.uk/chembl/. These data were derived from the following resources available in the public domain: ChEMBL, https://www.ebi.ac.uk/chembl/.
